# Protocol: Proactive resilience programmes for improving resilience and psychological adaptation in employees in high‐risk occupations: A systematic review

**DOI:** 10.1002/cl2.70007

**Published:** 2024-11-22

**Authors:** Trine Filges, Anja Bondebjerg Mølgaard, Geir Smedslund, Malene W. Kildemoes, Elizabeth Bengtsen

**Affiliations:** ^1^ VIVE Campbell VIVE – The Danish Center for Social Science Research Copenhagen Denmark; ^2^ Norwegian Medical Products Agency Oslo Norway

**Keywords:** high‐risk occupation, meta‐analysis, resilience

## Abstract

This is the protocol for a Campbell systematic review. The objectives are as follows: What are the effects of proactive resilience programmes offered to employees in high‐risk occupations on resilience and psychological adaptation?

## 1 BACKGROUND

### 1.1 The problem, condition, or issue

There is an increasing interest worldwide in promoting positive mental health due to its beneficial influence on not only educational attainment, productivity, employment, and earnings, but also social cohesion, engagement, and quality of life (Friedli & World Health Organization. Regional Office for Europe, 2009). Individuals employed in occupations associated with high stress levels and extreme conditions (such as the police, fire and rescue services, health and social services, and the military) commonly report stress‐related mental health conditions arising from their exposure to multiple traumas. Elevated rates of anxiety, depression, posttraumatic stress disorder (PTSD), and substance misuse and abuse have been documented in populations associated with high stress levels and extreme conditions (Hoell et al., 2023; Jones, 2017; Joyce et al., 2019; Matthews et al., 2022; Testa et al., 2023).

Higher levels of resilience have been shown to protect the long‐term mental health of first‐responders such as firefighters (Joyce et al., 2019). Thus, especially for individuals employed in occupations associated with high stress and extreme conditions is resilience important for the ability to continued functioning.

According to the APA (Dictionary of Psychology: https://dictionary.apa.org/resilience), resilience is: ‘the process and outcome of successfully adapting to difficult or challenging life experiences, especially through mental, emotional, and behavioural flexibility and adjustment to external and internal demands. A number of factors contribute to how well people adapt to adversities, predominant among them (a) the ways in which individuals view and engage with the world, (b) the availability and quality of social resources, and (c) specific coping strategies. Psychological research demonstrates that the resources and skills associated with more positive adaptation (i.e., greater resilience) can be cultivated and practiced’. Thus, resilience is thought to be modifiable (Southwick, 2011) and as such amenable to intervention. In the present review, we therefore aim to analyse the effects of interventions targeting resilience delivered within the workplace.

When evaluating the effect of interventions to improve resilience, an important distinction should be made between the *capacity for* resilience and the *demonstration* of resilience. The capacity for resilience addresses factors associated with the potential ability of an individual to show positive adaption in the face of stressors or significant adversity (Masten, 2001) whereas demonstration of resilience refers to the documentation that individuals who have experienced significant adversity have exhibited successful adaptation (Bonanno, 2004). More specifically, without the experience of stressors, adversity, or hardship, there is nothing to be resilient against (Fisher, 2019). Restricting the eligible occupations to high‐risk occupations, that is, workplaces where employees are likely to be exposed to what would be considered significant adversity has the advantage of not only enabling the documentation of capacity for resilience but also the demonstration of resilience (Britt et al., 2016).

#### 1.1.1 Significant adversity

A relevant question is: What constitutes significant adversity? We will not come up with a firm definition, instead we will give examples of what is eligible in this review and what is not.

Exposure to potentially traumatic events that occur within the context of an occupation certainly constitute significant adversity.

Trauma, according to DSM‐5, is defined as exposure to actual or threatened death, serious injury or sexual violence in one or more of four ways: (1) directly experiencing the event; (2) witnessing, in person, the event occurring to others; (3) learning that such an event happened to a close family member or friend; and (4) experiencing repeated or extreme exposure to aversive details of such events, such as with first responders. Actual or threatened death must have occurred in a violent or accidental manner; and experiencing cannot include exposure through electronic media, television, movies or pictures, unless it is work‐related. Note that the fourth type, Repeated or extreme exposure to details of the event(s), that is, vicarious trauma, is an amendment to the DSM‐5, which retained all three types of exposure from the DSM‐IV/‐TR and added a fourth. Further, such exposure through media, television, movies or pictures does not qualify unless for work.

In addition, chronic exposure to particularly work‐related intense stressors, such as sexual harassment, abusive supervision, or potentially physical stressors (e.g., extreme heat/cold, lack of sleep and rest, unhygienic conditions or crowding), also represents significant adversity (Fikretoglu & McCreary, 2012). As opposed to the above mentioned intense stressors, we will argue that many of the traditional work stressors examined by organisational psychologists, including job ambiguity, work overload, and organisational constraints (Britt et al., 2016), do not constitute significant adversity.

### 1.2 Policy relevance

Due to its potential influence on mental health, resilience is of special interest from both a policy and a practice standpoint (Windle et al., 2011). For employees in high‐risk occupations with an exposure to significant adversity, resilience is vital for their ability to continued functioning and avoidance of mental health problems. Mental health problems may be long‐lasting and can have a profound influence on an individual's daily life. The present review will provide further and more detailed evidence on the effect of proactive resilience interventions offered to employees exposed to significant adversity during their line of work. Policy makers as well as practitioners and managers working with or experiencing significant stressors within their workplace may benefit from the knowledge generated by the present work.

### 1.3 The intervention

The eligible interventions are resilience interventions given individually, in a group or at the organisational level. Resilience interventions can be any programme explicitly designed to develop, enhance, or improve resilience.

However, there is little consensus as to when a programme may be considered a resilience programme, just as there is no clear definition of what constitute the necessary components of an effective resilience programme (Joyce et al., 2018; Leppin et al., 2014). There is wide diversity across resilience programmes in the operationalisation of the construct, and in which core components are included (Joyce et al., 2018; Kunzler et al., 2020a).

According to Reivich et al. (2011), psychologists have been studying resilience since the 1970s, and research has demonstrated that there are many aspects of resilience that are teachable.

Programmes may focus on fostering a single or multiple psychosocial resilience factors.

According to American Psychological Association (2020), the modifiable behaviours, thoughts, and actions to foster resilience are: prioritise relationships, foster wellness (e.g., take care of your body [nutrition, sleep, regular exercise], practice mindfulness and avoid negative outlets such as alcohol and drugs), find purpose (e.g., help others, be proactive, move toward your goals and look for opportunities for self‐discovery) and embrace healthy thoughts (e.g., keep things in perspective, accept change, maintain a hopeful outlook and learn from your past).

Currently, however, no empirically validated theoretical framework exists that outlines which resources and skills to target nor the mode of action of resilience interventions.

As an example, the Penn Resilience Programme was developed at the University of Pennsylvania in 1990 as a school‐based training programme for students in late childhood and early adolescence. The programme was modified for a military student population in 2009 and is now part of the Comprehensive Soldier Fitness programme (Cornum et al., 2011; Reivich et al., 2011). The resilience training programme focuses on a subset of the factors identified by research, primarily the research by Masten and Reed (Masten & Reed, 2002; Masten, 2001). These factors include optimism, problem solving, self‐efficacy, self‐regulation, emotional awareness, flexibility, empathy, and strong relationships, which are, to some extent, overlapping with the modifiable factors emphasised by the APA.

Systematic reviews on resilience interventions (Joyce et al., 2018; Kunzler et al., 2020a) find that most interventions are based on psychotherapeutic approaches such as cognitive‐behavioural therapy (CBT), mindfulness, and variants or combinations of these such as acceptance and commitment therapy, attention and interpretation therapy, problem solving therapy and stress inoculation.

### 1.4 How the intervention might work

Resilience is not only a personality trait that only some people possess, although certain factors might make some individuals more resilient than others. On the contrary, resilience involves behaviours, thoughts, and actions that anyone can learn and develop. Resilience interventions might work by strengthening these factors.

Two overarching theoretical foundations of resilience interventions which have been identified in systematic reviews (Doody et al., 2021; Joyce et al., 2018; Kunzler et al., 2020a) are CBT‐based and mindfulness‐based (and combinations).

CBT is a multicomponent intervention that combines cognitive and behavioural therapy (Beck, 2011). While *cognitive therapy* is concerned with internal cognitive processes, *behavioural therapy* mainly focuses on external settings and observable behaviour. CBT is based on the premise that psychological problems are based, in part, on faulty or unhelpful ways of thinking and in part, on learned patterns of unhelpful behaviour and through therapy, people can learn better ways of coping with these problems, thereby relieving their symptoms and becoming more effective in their lives (American Psychological Association, 2024). CBT treatment usually involves efforts to change thinking patterns and change behavioural patterns.

Mindfulness can be traced back to Buddhist spiritual traditions, in which it is viewed as a key practice for becoming aware of oneself (Musil et al., 2021). Mindfulness approaches seek to improve psychosocial well‐being by helping individuals become aware of thoughts and feelings and recognise how those thoughts and feelings are connected to one's body and experiences (Bishop et al., 2004; Samuel, 2015).

### 1.5 Why it is important to do this review

In the present review, we aim to analyse effects of resilience interventions offered to employees who are exposed to what would be considered significant adversity during their line of work.

We have located four systematic reviews with meta‐analysis on resilience training programmes for adults considered at risk due to their occupation, although one of these includes studies without a control group.

Kunzler et al. (2020a) performed a systematic review to assess the effects of interventions to foster resilience in healthcare professionals, that is, healthcare staff delivering direct medical care (e.g., nurses, physicians, hospital personnel) and allied healthcare staff (e.g., social workers, psychologists). The eligible interventions focused on fostering resilience or the related concepts of hardiness or post‐traumatic growth by strengthening well‐evidenced resilience factors that are thought to be modifiable by training. Randomised controlled trials (RCTs) in adults aged 18 years and older who are employed as healthcare professionals were eligible. Initially, it was planned to include clinical and non‐clinical populations (e.g., patients, employees, students, military), but due to a large number of studies located it was decided to narrow the eligible population to healthcare professionals and healthcare students, the latter being analysed in an independent review (Kunzler et al., 2020b). The primary outcomes considered were resilience, anxiety, depression, stress or stress perception, and well‐being or quality of life. Secondary outcomes were resilience factors. The search was performed from 1990 to June 2019. Forty‐four RCTs were included in Kunzler et al. (2020a) and 30 studies were included in Kunzler et al. (2020b). The overall conclusion was a very low certainty evidence that resilience training may result in higher levels of resilience, lower levels of depression, stress or stress perception, and higher levels of certain resilience factors at post‐intervention.

For healthcare students, there was very low certainty evidence for the effect of resilience training on resilience, anxiety, and stress or stress perception at post‐intervention.

Joyce et al. (2018) analysed any programme designed to develop, enhance, or improve resilience in adults. No eligibility criteria for participants were specified. RCT and non‐randomised controlled trials (NRCTs) were eligible as long as the study described a specific aim to improve resilience and employed an acceptable measure of resilience as one of the outcome measures. The search was performed up to July 2016 and 11 studies were included in the meta‐analysis. This is a surprisingly low number of studies given the lack of population eligibility criteria but may be due to the rather simple, limited search strategy used to identify relevant studies. For example, the search strategy used for the Medline database was ‘Resilience *and/or* resiliency *and* resilience training *and/or* resilience intervention’, which produced only 169 hits. In comparison, the more comprehensive search strategy used in Kunzler et al. (2020a) resulted in 6723 hits in the Medline database even though it was restricted to studies from 1990 to 2016 (p. 349). Although a meta‐analysis was performed in Joyce et al. (2018), it most likely is not representative due to the narrow search strategy and limited number of studies located.

Vanhove et al. (2015) analysed studies of resilience‐building programmes implemented in organisational contexts. No participant eligibility criteria were specified. Eligible interventions were preventive interventions; interventions or therapies such as stress debriefing, which aimed to treat existing problems associated with specific past traumas or exposures to stress, were not eligible, and neither were pure stress management interventions. RCTs, non‐randomised studies (NRS), as well as before‐after (BA) studies were eligible. The search strategy was rather narrow. Only one electronic database (PsycINFO) and in addition a restricted electronic database (Google Scholar) were searched, using combinations of the three sets of search terms: ‘resilience’ and ‘resiliency’; ‘intervention’, ‘program’, and ‘training’; and finally, ‘work’, ‘organization’, and ‘employee’. The electronic database searches up to 2014 returned a total of 1411 articles. In addition, the authors performed reference screening of both primary studies and reviews. Thirty‐seven primary studies, of which 8 were BA, were included. Resilience outcomes were not reported. In the meta‐analysis, authors aggregated the full range of psychosocial health and performance‐related outcomes included in primary studies into one outcome (method not reported) and also included the effects obtained from BA studies. In addition, results separated by time (post and follow‐up) and by three outcome categories: performance (e.g., supervisor‐rated performance, successful task completion), psychological deficits (e.g., anxiety, depression), and wellbeing (e.g., positive affect, purpose in life, subjective well‐being) were reported. In total, 342 separate effect sizes were reported in the included studies, that is, also in the analyses separated by time and outcome category were aggregated outcome measures used.

We further located one systematic review (Di Nota et al., 2021) with meta‐analysis, although it included a broad variety of proactive psychological programme types and not just programmes on resilience‐building and included studies without a control group. The aim of the review was to investigate the degree to which various occupationally mediated posttraumatic stress injuries were impacted by different programming approaches. The different training programmes were categorised into: Prevention, Resilience, Coping (skills), Family coping, Stress reduction, Skill building, Wellness capacity, Capacity building, Psychoeducation, Mental health awareness (training), and Stigma reduction. The eligible participants were adult workers (aged 18 and older) exposed to potentially psychologically traumatic events, including counsellors, correctional workers, dispatchers, emergency workers, firefighters, nurses, paramedics, police, rail transit operators, and social workers. RCTs, NRS, as well as BA studies were eligible. The searches were restricted to peer‐reviewed English‐ or French‐language studies, published between 2008 and December 9, 2019.

Forty‐two primary studies, of which 10 were BA, were included. 36 studies were included in a quantitative meta‐analysis. Effect sizes from studies of all types of designs (i.e., RCTs and NRS with control group and BA without control group) were pooled. The type of effect size used for the studies with control group was not reported; that is, if between group effect sizes or within group effect sizes were used (from studies with control group) and pooled with within group effect sizes from the BA studies.

We located six systematic reviews without meta‐analysis on resilience training programmes for adults considered at risk due to occupation and one systematic review without meta‐analysis on a broader variety of psychological and well‐being programmes, including programmes on resilience‐building.

Ferreira et al. (2021) systematically searched for studies of interventions aimed at enhancing, promoting, or developing individual resilience in adults (>18 years). A narrow search strategy along the lines of Vanhove et al. (2015) was performed in four electronic databases (Science Direct, B‐on, PsycNet and PubMed) for studies published between 2010 and 2020. RCTs and NRSs reporting a valid and appropriate measure of resilience were eligible. The included studies were grouped according to potential risk source of which only the group ‘considered at risk due to occupation’ is relevant for the current review (other groups were general population, at risk due to clinical condition, and at risk due to caretaking). In total, 38 studies were included of which 16 were categorised as a group considered at risk due to occupation.

Macedo et al. (2014) systematically searched for studies of training programmes aimed to strengthen resilience to prepare individuals from non‐clinical adult samples to cope with future adverse events. Programmes that focused on strengthening resilience in physically and/or mentally ill individuals were not eligible, nor were programmes with a primary focus on assessing resilience in the aftermath of exposure to specific traumatic events (e.g., resilience training after a natural disaster). Three electronic databases were searched (ISI, PsycINFO, and PubMed). RCTs and NRSs reporting a ‘standardized efficacy measure’ were eligible. The search was performed up to January 2013 and 13 studies were included.

Doody et al. (2021) systematically searched for studies of any intervention designed to build pre‐deployment resilience in military or emergency service personnel. A comprehensive search was performed in several electronic databases up to September 2020 and further grey search and screening of reference lists was performed. Only RCTs were eligible. Twenty‐eight studies were included (15 including military personnel and 13 including emergency workers). A narrative synthesis was presented due to the level of heterogeneity identified in the studies.

Thompson and Dobbins (2018) performed a restricted search for studies published between 2006 and 2016 in peer‐reviewed journals. The eligible population was active duty military personnel, with further eligibility requirements specifying that subjects had to be English speaking adults ages 18 and older. Interventions should have a focus on resilience or stress management. Although only RCTs were eligible, one NRCT study was included. In total, five studies were included.

Robertson et al. (2015) searched for studies of workplace resilience interventions published between 1989 and 2013. The eligible population was employees aged 18 years or older. A very narrow search strategy conducted in the Cochrane Central Register of Controlled Trials, MEDLINE, and PsycINFO was performed and resulted in only 155 hits. In total, 14 studies were included, 8 RCTs, 2 NRCTs, and 4 BAs. A narrative synthesis was presented.

Skeffington et al. (2013) systematically searched for studies of resilience‐building programmes delivered to populations in any country, in any year, and of any age who were subsequently exposed to a potentially traumatic event. The event was required to meet Criterion A‐1 for PTSD outlined in the DSM–IV (American Psychiatric Association, 1994). The searches were performed up to November 25, 2011, and resulted in seven included studies of which five were NRCT and two were single‐group post‐only studies (i.e., a pre‐post ‘effect size’ could not be calculated). A narrative synthesis was presented.

Wild et al. (2020) systematically searched for studies on interventions for first responders, which focus on improving well‐being, resilience, or stress management. The eligible evaluations should be published in peer‐reviewed studies published between 1980 and 2018. Eligible participants were adults aged 18 years or above, currently working within the emergency services (ambulance, fire, first aid responders, police, and search and rescue).

Thirteen studies were included. The authors did not perform a meta‐analysis, only a narrative synthesis based on both within group and between group effect sizes. The within group effect sizes were calculated assuming statistical independence for pre‐ and post‐intervention, that is, uncorrelated measures. Further, they only calculated between‐group effect sizes if there were no significant differences at baseline, that is, a very selected group of studies. The review did not discriminate between interventions aimed at improving well‐being, resilience, or stress management.

To sum up, 10 systematic reviews have investigated the efficacy of resilience interventions in adults, and in addition, 2 reviews investigated a broader variety of proactive psychological and well‐being programmes, including programmes on resilience‐building. Of these two latter reviews, one did not perform a meta‐analysis (Wild et al., 2020) and the one that did perform a meta‐analysis included BAs in the meta‐analysis (Di Nota et al., 2021).

However, all 10 publications on resilience interventions are either narrowly restricted in the eligible population or perform a very narrow search. In addition, to date, only Joyce et al. (2018), Kunzler et al. (2020a, 2020b), and Vanhove et al. (2015) were able to perform a meta‐analysis of which one (Vanhove et al., 2015) included BAs.

We will perform a review with a wider eligible population and a comprehensive search.

We seek to identify gaps in existing knowledge to enhance future options for prioritising research in the field. This review is also conducted to inform potential next steps in policy development in the area of employees exposed to significant adversity. By identifying the major effects of proactive resilience interventions and quantifying these effects, the review can inform policy development and may enable decision makers to prioritise key areas.

## 2 OBJECTIVES

What are the effects of proactive resilience programmes offered to employees in high‐risk occupations on resilience and psychological adaptation?

## 3 METHODS

The proposed project will follow standard procedures for conducting systematic reviews using meta‐analytic techniques.

### 3.1 Criteria for considering studies for this review

#### 3.1.1 Types of studies

The study designs we will include in the review are:

A. Controlled trials (where all parts of the study are prospective, such as identification of participants, assessment of baseline, and allocation to intervention, and which may be randomised or non‐randomised, assessment of outcomes and generation of hypotheses (Higgins & Green, 2011)).
B. Quasi‐experimental studies (attendance in programmes has occurred in the course of usual decisions, the allocation to programmes or no programme is not controlled by the researcher, and there is a comparison of two or more groups of participants, i.e., at least a treated group and a control group).

To summarise what is known about the possible causal effects of programme participation, we will include all study designs that use a control group. Controlled trials, whether randomised or non‐randomised, will be included. Quasi‐experimental studies (NRS), where attendance in the programmes has occurred in the course of usual decisions outside the researcher's control, must demonstrate pre‐treatment group equivalence via matching, statistical controls, or evidence of equivalence on key risk variables and participant characteristics. Pre‐treatment group equivalence factors are outlined in Section 3.3.4, and the methodological appropriateness of the included studies will be assessed according to a risk of bias model.

Studies using single group pre‐post comparisons will not be included. Furthermore, studies using single group pre‐post comparisons and NRS using an instrumental variable approach will not be included—see the Supporting Information: Appendix (Justification of exclusion of studies using an instrumental variable (IV) approach) for our rationale for excluding studies with these designs. A further requirement to all types of studies (randomised as well as non‐randomised) is that they are able to identify an intervention effect. Studies where, for example, the treatment is given to one single organisation/company/workplace/department only and the comparison group is another organisation/company/workplace/department (or more organisations/companies/workplaces/departments for that matter) cannot separate the treatment effect from the organisation/company/workplace/department effect. Therefore, there must be at least two organisations/companies/workplaces in both the treatment and comparison group.

Special caution will be taken concerning studies using regression discontinuity designs (RDD) to estimate the treatment effect. In sharp RDDs, a threshold of a (non‐manipulable) forcing/running variable determines which workplaces receive a treatment and which do not, that is, the design is similar to an RCT in the sense that the random sequence determining treatment assignment can be seen as a running variable (Lee & Lemieux, 2008). In contrast, in ‘fuzzy’ RDDs, being on one side of a threshold only makes it more likely that a workplace ends up in the treatment or control group, and the threshold is used as an instrument to estimate local average treatment effects (LATE) (Imbens & Lemieux, 2008). That is, fuzzy RDD is a form of IV analysis, which we will exclude due to the comparability issues mentioned earlier. Sharp RDDs will be included.

We will not include qualitative research.

#### 3.1.2 Types of participants

The eligible population is employees in high‐risk occupations such as the police, fire and rescue services, health and social services, the military and frontline reporters. Note this is not necessarily an exhaustive list of eligible occupations, others may appear during the search that we have not thought about. Employees should be aged 18 years or older. We will include studies involving employees less than 18 years of age, as well as those aged 18 years and older, if data for participants aged 18 years and above are reported separately or can be obtained by contacting the study authors.

#### 3.1.3 Types of interventions

The eligible interventions are resilience interventions offered to eligible employees as defined above. The programmes may be given individually in a group or at the organisational level. Resilience interventions can be any programme explicitly designed to develop, enhance, or improve resilience in adults. Interventions where fostering resilience is not explicitly formulated as an aim are not eligible.

Only proactive interventions, intended to promote the likelihood of resilience in response to potential future occurrences of adversity, are eligible. Programmes aimed at treating existing problems associated with specific past traumas or exposures to stress, such as stress debriefing, are not eligible.

#### 3.1.4 Types of outcome measures

##### Primary outcomes

We will only include outcomes at the individual level, although training may be given at the group or organisational level.

The primary outcomes are resilience measurement scales and other assessments of psychological adaptation (e.g., mental health).

The resilience measures have to assess an individual's ability to adapt and cope effectively with significant adversity and must have undergone some type of validity assessment. The review by Windle et al. (2011) has systematically reviewed the psychometric rigour of resilience measurement scales developed for use in general and clinical populations. Unfortunately, the conclusion of the review is that there is currently no gold standard measure of resilience, but the following seven measures are examples of outcomes that meet our criteria:

− The Dispositional Resilience Scale (Bartone, 1991, 1995, 2007).
− The Connor‐Davidson Resilience Scale (CDRISC) (Connor & Davidson, 2003).
− The Resilience Scale for Adults (RSA) (Friborg et al., 2003, 2005).
− The Resilience Scale (RS) (Wagnild & Young, 1993).
− Psychological Resilience (Windle et al., 2008).
− Ego Resiliency (Klohnen, 1996).
− The Brief Resilience Scale (Smith et al., 2008).

Mental health will be divided into four categories:

− Post‐traumatic stress, that is, post‐traumatic stress symptoms including secondary traumatic stress symptoms/reactions and PTSD.
− Anxiety symptoms.
− Depressive symptoms.
− Stress including occupational stress.

The mental health outcomes shall be measured by standardised psychological symptom measures as, for example, the PTSD Checklist (PCL), the Harvard Trauma Questionnaire, the Impact of Events Scale‐revised (IES‐R), the Secondary Traumatic Stress Scale (STSS), the Hopkins Symptom Checklist, the Hospital Anxiety and Depression scale (HADS (D and A)), the Depression Anxiety and Stress Scales (DASS), the Beck Anxiety Inventory (BAI), the Beck Depression Inventory (BDI), the Perceived Stress Questionnaire (PSQ), Occupational Stress Inventory (OSI) and Stress Response Inventory (SRI).

If the prevalence of a diagnosis of either of the three mental health outcomes (PTSD, any anxiety disorder or depression) is reported, they will be analysed separately.

A *diagnosis* refers to the justifiable conclusion made by a regulated health care professional within their scope of practice, usually using the criteria outlined in the Diagnostic and Statistical Manual of Mental Disorders (DSM) or International Classification of Diseases (ICD) (see Heber et al., 2023).

##### Secondary outcomes

Secondary outcomes are alcohol and substance abuse measured by self‐report, by reports from authorities, derived from administrative files or registers, or from results of biochemical tests for drug and alcohol use.

##### Duration of follow‐up

Time points for measures considered will be:

• post and short‐term follow‐up (up to 3 months);

• medium‐term follow‐up (4–12 months); and

• long‐term follow‐up (over 12 months).

##### Types of settings

All settings such as multimedia programmes or face‐to‐face settings, and delivered in a group or individual context are eligible.

### 3.2 Search methods for identification of studies

Relevant studies will be identified through electronic searches in bibliographic databases, grey literature repositories and resources, hand search in specific targeted journals, citation tracking, contact with international experts and Internet search engines. A date restriction of 1990 and onwards will be applied, as the idea of promoting resilience by specific training is relatively new (Kunzler et al., 2020a).

#### 3.2.1 Electronic searches

The following electronic bibliographic databases will be searched:

- ERIC (EBSCO).
- Academic Search Premier (EBSCO).
- MEDLINE (OVID).
- Embase (OVID).
- CINAHL (EBSCO).
- Cochrane Library (Cochrane Reviews & Cochrane Central).
- PsycINFO (EBSCO).
- APA PsycNet.
- SocINDEX (EBSCO).
- International Bibliography of the Social Sciences (ProQuest).
- Sociological Abstracts (ProQuest).
- Science Citation Index Expanded (Web of Science).
- Social Sciences Citation Index (Web of Science).

We have consulted the list of databases comprised in the article by Kugley et al. (2017).

##### Description of the search‐string

The search string is based on the PICO(s)‐model, and contains three concepts, of which we have developed three corresponding search facets: population characteristics, the intervention and study design. The search string includes searches in title and abstract as well as subject terms and/or keywords for each facet. The subject terms in the facets will be selected according to the thesaurus or index of each database. Keywords will be supplied if the search technique provides additional results. Use of truncation and wildcards will be used to address English spelling variants.

##### Example of a search‐string

The search string below is developed to search MEDLINE through the OVID search interface and exemplifies the search facets as they will be searched:

**Table:** 

**#**	**Query**
1	(‘first responder*’ or ‘front line worker*’ or ‘frontline worker*’ or ‘frontline responder*’ or ‘frontline responder*’ or ‘front line employee*’ or ‘frontline employee*’ or ‘frontline personnel*’ or ‘front line personnel*’ or journalist* or ‘news* reporter*’).ti. or (‘first responder*’ or ‘front line worker*’ or ‘frontline worker*’ or ‘frontline responder*’ or ‘frontline responder*’ or ‘front line employee*’ or ‘frontline employee*’ or journalist* or ‘news* reporter*’).ab. or (emergency adj (worker* or services or responder* or personnel or crew or crews)).ti. or (emergency adj (worker* or services or responder* or personnel or crew or crews)).ab.
2	limit 1 to yr = ‘1990–2024’
3	Emergency Responders/
4	limit 3 to yr = ‘1990–2024’
5	(paramedic* or physician* or ambulancem#n or police* or military or ‘fire fighter*’ or firefighter* or firem#n or ‘law enforcement personnel’ or ‘social services’ or nurse*).ti. or (paramedic* or physician* or ambulancem#n or police* or military or ‘fire fighter*’ or firefighter* or firem#n or ‘law enforcement personnel’ or ‘social services’ or nurse*).ab. or ((‘health care’ or healthcare or rescue or ambulance) adj3 (professional* or personnel or provider* or services or staff or ‘worker*))).ti. OR (((health care OR healthcare OR rescue OR ambulance) ADJ3 (professional* OR personnel OR provider* OR services OR staff OR worker*’)).ab.
6	limit 5 to yr = ‘1990–2024’
7	emergency medical technicians/or combat medics/or firefighters/or paramedics/or police/or *health personnel/or military personnel/
8	limit 7 to yr = ‘1990–2024’
9	2 or 4 or 6 or 8
10	(Resilien* or hardiness* or ‘stress immunity’ or ‘post‐traumatic growth’ or ‘posttraumatic growth’ or ‘stress‐related growth’ or ‘post‐traumatic stress’).ti. or (Resilien* or hardiness* or ‘stress immunity’ or ‘post‐traumatic growth’ or ‘posttraumatic growth’ or ‘stress‐related growth’ or ‘post‐traumatic stress’).ab. or ((social adj1 (adapt* or adjust*)) or (positiv* adj1 (adapt* or adjust*)) or (psychol* adj1 (adapt* or adjust*)) or (cope or coping) or ((withstand* or overcom* or resist* or recover* or thriv* or adapt* or adjust*) adj3 (stress* or trauma* or adversit*))).ti. or ((social adj (adapt* or adjust*)) or (positiv* adj (adapt* or adjust*)) or (psychol* adj (adapt* or adjust*)) or (cope or coping) or ((withstand* or overcom* or resist* or recover* or thriv* or adapt* or adjust*) adj3 (stress* or trauma* or adversit*))).ab.
11	limit 10 to yr = ‘1990–2024’
12	posttraumatic growth, psychological/or resilience, psychological/or adaptation, psychological/or coping skills/or emotional adjustment/
13	limit 12 to yr = ‘1990–2024’
14	(psychotherap* or ‘psycho‐therap*’ or (behave* adj3 (intervention* or program* or therap*)) or ((cognit* or ‘cognitive behavi*’ or ‘CBT’) adj3 (intervention* or program* or therap*)) or (psycho* adj3 (intervention* or program* or therap*)) or relaxation or mindful* or (counsel?ing or coaching) or (‘third wave’ adj (psycho* or therap*)) or ‘cognit* restructure*’ or ‘positive psychology’ or (stress adj (inoculation or manag* or reduc* or resist*)) or (anxiety adj3 manage*) or ‘acceptance and commitment’ or (health adj3 (educat* or promot*))).ti. or (psychotherap* or ‘psycho‐therap*’ or (behave* adj3 (intervention* or program* or therap*)) or ((cognit* or ‘cognitive behavi*’ or ‘CBT’) adj3 (intervention* or program* or therap*)) or (psycho* adj3 (intervention* or program* or therap*)) or relaxation or mindful* or (counsel?ing or coaching) or (‘third wave’ adj (psycho* or therap*)) or ‘cognit* restructure*’ or ‘positive psychology’ or (stress adj (inoculation or manag* or reduc* or resist*)) or (anxiety adj3 manage*) or ‘acceptance and commitment’ or (health adj3 (educat* or promot*))).ab.
15	limit 14 to yr = ‘1990–2024’
16	Psychotherapy/or cognitive behavioural therapy/or ‘acceptance and commitment therapy’/
17	limit 16 to yr = ‘1990–2024’
18	15 or 17
19	11 or 13
20	18 and 19
21	((resilien* adj5 (train* or program* or intervention* or promot* or prevent* or enhance* or learn* or teach* or educat* or increase* or develop* or manag* or therap* or protocol* or treat*)) or (hardiness* adj5 (train* or program* or intervention* or promot* or prevent* or enhance* or learn* or teach* or educat* or increase* or develop* or manag* or therap* or protocol* or treat*))).ti. or ((resilien* adj5 (train* or program* or intervention* or promot* or prevent* or enhance* or learn* or teach* or educat* or increase* or develop* or manag* or therap* or protocol* or treat*)) or (hardiness* adj5 (train* or program* or intervention* or promot* or prevent* or enhance* or learn* or teach* or educat* or increase* or develop* or manag* or therap* or protocol* or treat*))).ab.
22	limit 21 to yr = ‘1990–2024’
23	20 or 22
24	(effect* or trial* or experiment* or control* or random* or impact* or compar*).ti. or (effect* or trial* or experiment* or control* or random* or impact* or compar*).ab.
25	limit 24 to yr = ‘1990–2024’
26	clinical trial/or randomised controlled trial/or Research Design/
27	limit 26 to yr = ‘1990–2024’
28	25 or 27
29	9 and 23 and 28

#### 3.2.2 Searching other resources

##### Hand‐search

We will conduct a hand search of specific journals, to make sure that all relevant articles are found. The hand search will focus on editions published between 2022 and 2024 to secure recently unpublished articles which have not yet been indexed in the bibliographic databases.

We will decide upon which journals to hand search based on the identified records from the electronic searches. The following are examples of specific journals which we may decide to hand search:

- *International Journal of Stress Management.*
- *Journal of Occupational and Environmental Medicine.*
- *Military Medicine.*
- *Psychiatry Research.*
- *Journal of Primary Prevention.*
- *Workplace Health & Safety.*

##### Grey literature searches

We will search for such references as dissertations, working papers, reports, and evidence and gap maps (EGMs) or systematic reviews. Most of the resources searched may include multiple types of references, both published and unpublished. In general, there is a great amount of overlap between the types of references in the chosen resources. The resources are listed once under the category of literature we expect to be most prevalent in the resource, even though multiple types of unpublished/published literature might be identified in the resource. A final list of resources will be included in an appendix.

##### Dissertations

We will search the following resources for dissertations:

- Open Access Theses and Dissertations.
- EBSCO Open Dissertations (EBSCO‐host).

##### Working papers

We will search the following resources for working papers:

- Google Scholar: https://scholar.google.com/.
- Social Science Research Network: https://www.ssrn.com/index.cfm/en/.

For each resource, we will screen the first 100 hits.

##### EGMs and systematic reviews

To locate any potential EGMs or systematic reviews, we will search the following resources:

- Campbell Systematic Reviews Journal: https://onlinelibrary.wiley.com/journal/18911803?af=R.
- EPPI‐Centre publications: https://eppi.ioe.ac.uk/cms/Default.aspx?tabid=116.
- PROSPERO: https://www.crd.york.ac.uk/prospero/.
- Epistemonikos: https://www.epistemonikos.org/.

If we identify relevant systematic reviews or EGMs during the search process, they will be used for citation‐tracking.

##### Trial registries

- CENTRAL Trials Register within the Cochrane Library: https://www.cochranelibrary.com/central (includes ClinicalTrials.gov: https://clinicaltrials.gov/ and WHO International Clinical Trials Registry Platform: https://www.who.int/clinical-trials-registryplatform.

### 3.3 Data collection and analysis

#### 3.3.1 Description of methods used in primary research

RCTs are eligible, and we expect that most studies will be conducted with randomisation of participants. Studies conducted without randomisation of participants are eligible but will be required to have a control group for inclusion in the review. Participants may be allocated by, for example, location differences, decision makers, policy rules or participant preferences. We expect that the primary studies demonstrate pre‐treatment group equivalence via matching, statistical controls, or evidence of equivalence on key risk variables and participant characteristics as outlined in Section 3.3.4.

#### 3.3.2 Selection of studies

Under the supervision of review authors, two review team assistants will first independently screen titles and abstracts to exclude studies that are clearly irrelevant. Studies considered eligible by at least one assistant or studies were there is insufficient information in the title and abstract to judge eligibility, will be retrieved in full text. The full texts will then be screened independently by two review team assistants under the supervision of the review authors. Any disagreement of eligibility will be resolved by the review authors. Exclusion reasons for studies that otherwise might be expected to be eligible will be documented and presented in an appendix. The study inclusion criteria will be piloted by the review authors and team assistants (see Supporting Information: Appendix 2). The overall search and screening process will be illustrated in a flow diagram. None of the review team members will be blind to the authors, institutions, or the journals responsible for publication of the articles.

#### 3.3.3 Data extraction and management

Two review authors will independently code and extract data from all included studies. A coding sheet will be piloted on several studies and revised as necessary (see Supporting Information: Appendix 3). Disagreements will be resolved by consulting a third review author with extensive content and methods expertise. Disagreements resolved by a third reviewer will be reported. Data and information will be extracted on: available characteristics of participants, intervention characteristics and control conditions, research design, sample size, risk of bias and potential confounding factors, outcomes, and results. Extracted data will be stored electronically. Analysis will be conducted using RevMan 5.4.1, Stata 18 and R software.

#### 3.3.4 Assessment of risk of bias in included studies

We will assess the risk of bias in randomised studies using Cochrane's revised risk of bias tool, ROB 2 (Higgins et al., 2019). The tool is structured into five domains, each with a set of signalling questions to be answered for a specific outcome. The five domains cover all types of bias that can affect results of randomised trials.

The five domains for individually randomised trials are:

(1) bias arising from the randomisation process;
(2) bias due to deviations from intended interventions (separate signalling questions for effect of assignment and adhering to intervention);
(3) bias due to missing outcome data;
(4) bias in measurement of the outcome;
(5) bias in selection of the reported result.

For cluster‐randomised trials, an additional domain is included ([1b] Bias arising from identification or recruitment of individual participants within clusters). We will use the latest template for completion (currently, it is the version of 15 March 2019 for individually randomised parallel‐group trials and 20 October 2016 for cluster randomised parallel‐group trials). In the cluster randomised template however, only the risk of bias due to deviation from the intended intervention (effect of assignment to intervention; intention to treat ITT) is present and the signalling question concerning the appropriateness of the analysis used to estimate the effect is missing. Therefore, for cluster randomised trials we will only use the signalling questions concerning the bias arising from identification or recruitment of individual participants within clusters from the template for cluster randomised parallel‐group trials; otherwise we will use the template and signalling questions for individually randomised parallel group trials.

We will assess the risk of bias in NRS, using the model ROBINS –I, developed by members of the Cochrane Bias Methods Group and the Cochrane Non‐Randomised Studies Methods Group (Sterne, Hernán, et al., 2016). We will use the latest template for completion (currently it is the version of 19 September 2016). The ROBINS‐I tool is based on the Cochrane RoB tool for randomised trials, which was launched in 2008 and modified in 2011 (Higgins et al., 2011). The ROBINS‐I tool covers seven domains (each with a set of signalling questions to be answered for a specific outcome) through which bias might be introduced into NRS:

(1) bias due to confounding;
(2) bias in selection of participants;
(3) bias in classification of interventions;
(4) bias due to deviations from intended interventions;
(5) bias due to missing outcome data;
(6) bias in measurement of the outcome;
(7) bias in selection of the reported result.

The first two domains address issues before the start of the interventions, and the third domain addresses classification of the interventions themselves. The last four domains address issues after the start of interventions and there is substantial overlap for these four domains between bias in randomised trials and bias in non‐randomised trials (although signalling questions are somewhat different in several places, see Sterne, Higgins, et al., 2016 and Higgins et al., 2019). Randomised study outcomes are rated on a ‘Low/Some concerns/High’ scale on each domain; whereas non‐randomised study outcomes are rated on a ‘Low/Moderate/Serious/Critical/No Information’ scale on each domain. The level ‘Critical’ means: the study (outcome) is too problematic in this domain to provide any useful evidence on the effects of intervention, and is therefore excluded from the data synthesis. The same critical level of risk of bias (excluding the result from the data synthesis) is not directly present in the RoB 2 tool, according to the guidance for the tool (Higgins et al., 2019).

In the case of an RCT, where there is evidence that the randomisation has gone wrong or is no longer valid, we will assess the risk of bias of the outcome measures using ROBINS‐I instead of ROB 2. Examples of reasons for assessing RCTs using the ROBINS‐I tool may include studies showing large and systematic differences between treatment conditions while not explaining the randomisation procedure adequately suggesting that there was a problem with the randomisation process; studies with large scale differential attrition between conditions in the sample used to estimate the effects; or studies selectively reporting results for some part of the sample or for only some of the measured outcomes. In such cases, differences between the treatment and control conditions are likely systematically related to other factors than the intervention and the random assignment is, on its own, unlikely to produce unbiased estimates of the intervention effects. Therefore, as ROBINS‐I allows for an assessment of, for example, confounding, it is more appropriate to assess effect sizes from studies with a compromised randomisation using ROBINS‐I rather than ROB 2 (Higgins et al., 2019 p. 14). If so, we will report this decision as part of the risk of bias assessment of the outcome measure in question. As other effect sizes assessed with ROBINS‐I, these effect sizes may receive a ‘Critical’ rating and thus be excluded from the data synthesis. We will stop the assessment of a non‐randomised study outcome as soon as one domain in the ROBINS‐I is judged as ‘Critical’. ‘Serious’ risk of bias in multiple domains in the ROBINS‐I assessment tool may lead to a decision of an overall judgement of ‘Critical’ risk of bias for that outcome, and it will be excluded from the data synthesis.

##### Confounding

An important part of the risk of bias assessment of NRS is consideration of how the studies deal with confounding factors. Systematic baseline differences between groups can compromise comparability between groups. Baseline differences can be observable (e.g., age and gender) and unobservable (to the researcher; e.g., motivation and ‘ability’). There is no single non‐randomised study design that always solves the selection problem. Different designs represent different approaches to dealing with selection problems under different assumptions, and consequently require different types of data. There can be particularly great variations in how different designs deal with selection on unobservables. The ‘adequate’ method depends on the model generating participation, that is, assumptions about the nature of the process by which participants are selected into a programme.

As there is no universal correct way to construct counterfactuals for non‐randomised designs, we will look for evidence that identification is achieved, and that the authors of the primary studies justify their choice of method in a convincing manner by discussing the assumption(s) leading to identification (the assumption(s) that make it possible to identify the counterfactual). Preferably the authors should make an effort to justify their choice of method and convince the reader that the only difference between a treated individual and a non‐treated individual is the treatment. The judgement is reflected in the assessment of the confounder unobservables in the list of confounders considered important at the outset (see Supporting Information: Appendix 4).

In addition to unobservables, we have identified the following observable confounding factors to be most relevant: age, gender, occupation, and resilience/mental health symptoms at baseline. In each study, we will assess whether these four indicators have been considered, and in addition we will assess other factors likely to be a source of confounding within the individual included studies.

The motivation for focusing on age, gender, occupation, and resilience/mental health symptoms at baseline is given below.

##### Importance of pre‐specified confounding factors

Resilience is increasingly being understood as the outcome of a dynamic process of successful adaptation to adversity, then, logically, resilience develops with age as the likelihood of having experienced adverse events or periods of difficult life circumstances increases with age (Kalisch, 2017). We therefore consider age to be a potential confounding factor.

Concerning gender, the nature of exposure may be different. For example, female service members, as compared to males, are likely to report a higher incidence of sexual trauma, including sexual assault and harassment while deployed (Murdoch et al., 2006). Further, given that some occupations are male‐dominated (e.g., the military) and others are female‐dominated (nursing), there may also be differential selection procedures by gender into the different occupations. Finally, early longitudinal studies of qualities that helped young people to be competent in the face of high‐risk environments found that being female is one of the resilient personal qualities identified (Rutter, 1985; Werner & Smith, 1992). There is thus the possibility that ‘true’ gender differences exist in the way that males and females process trauma exposure, leading to differential outcomes in the link between exposure and, for example, PTSD, although evidence is mixed (Street et al., 2009).

Different occupations face different levels of exposure to adversity and is therefore an important confounder.

Finally, pre‐treatment group equivalence of resilience or mental health symptoms is indisputable an important confounder. Therefore, the accuracy of the estimated effects of resilience programmes will depend crucially on how well pre‐treatment resilience or mental health symptoms are controlled for.

##### Effect of primary interest and important co‐interventions

We are mainly interested in the effect of starting and adhering to the intended intervention, that is, the treatment on the treated effect. The risk of bias assessments will therefore be in relation to this specific effect.

The risk of bias assessments of both randomised trials and nonrandomized studies will consider adherence and differences in additional interventions (‘co‐interventions’) between intervention groups. Relevant co‐interventions are those that individuals might receive with or after starting the intervention of interest and that are both related to the intervention received and prognostic for the outcome of interest. Important co‐interventions we will consider are any kind of mental health treatments delivered on an individual basis.

##### Assessment

At least two review authors will independently assess the risk of bias for each relevant outcome from the included studies. Any disagreements will be resolved by a third reviewer with content and statistical expertise and will be reported. We will report the risk of bias assessment in risk of bias tables for each included study outcome in the completed review.

#### 3.3.5 Measures of treatment effect

##### Continuous outcomes

For continuous outcomes, effects sizes in the form of standardised mean differences (SMDs) with 95% confidence intervals will be calculated, where means and standard deviations are available. If means and standard deviations are not available, we will calculate SMDs from *t*‐values, chi‐squared values and correlation coefficients, where available, using the methods suggested by Lipsey and Wilson (2001). If not, enough information is yielded, the review authors will request this information from the principal investigators. Hedges' *g* will be used for estimating SMDs. If there is a mix of studies with some reporting change scores and others reporting final values, we will contact authors and request the final values. If we do not obtain these values, we will analyse change scores and final values separately (Deeks et al., 2023, section 10.5.2). Any measures of resilience and mental health outcomes, are examples of relevant continuous outcomes in this review.

##### Dichotomous outcomes

For dichotomous outcomes, we will calculate odds ratios with 95% confidence intervals. Prevalence of any mental health diagnosis is an example of a relevant dichotomous outcome in this review. There are statistical approaches available to re‐express dichotomous and continuous data to be pooled together (Sánchez‐Meca et al., 2003). To calculate common metric odds ratios will be converted to SMD effect sizes using the Cox transformation. We will only transform dichotomous effect sizes to SMD if appropriate, for example, as may be the case if a study reports mental health symptoms as a dichotomous outcome. When effect sizes cannot be pooled, study‐level effects will be reported in as much detail as possible. Software for storing data and performing statistical analyses will be RevMan, Excel, R and Stata18.

#### 3.3.6 Unit of analysis issues

Errors in statistical analysis can occur when the unit of allocation differs from the unit of analysis. In cluster randomised trials, participants are randomised to treatment and control groups in clusters, either when data from multiple participants in a setting are included (creating a cluster within a workplace or organisation), or when participants are randomised by treatment locality or workplace. NRS may also include clustered assignment of treatment. Effect sizes and standard errors from such studies may be biased if the unit‐of‐analysis is the individual and an appropriate cluster adjustment is not used (Higgins & Green, 2011).

A study design where participants are individually allocated to treatment, but the treatment is delivered in a group setting, are known as individually randomised group treatment trials (Pals et al., 2008). The analysis in such a study design must also correct for the fact that dependencies may arise between individuals that happen to receive the intervention in the same group. If possible, we will adjust effect sizes individually using the methods suggested by Hedges (2007b) and information about the intra‐cluster correlation coefficient (ICC), realised cluster sizes, and/or estimates of the within and between variances of clusters. If it is not possible to obtain this information, we will adjust effect sizes using estimates from the literature (we will search for estimates of relevant ICC's), and assume equal cluster sizes. To calculate an average cluster size, we will divide the total sample size in a study by the number of clusters.

We will perform analyses separated by the time points suggested in Section 3.1.4.

#### 3.3.7 Criteria for determination of independent findings

To determine the independence of results in included studies, we will consider whether individuals may have undergone multiple interventions, whether there were multiple treatment groups, whether several studies are based on the same data source and whether studies report multiple conceptually similar outcomes.

##### Multiple interventions groups and multiple interventions per individuals

Studies with multiple intervention groups with different individuals will be included in this review, although only intervention and control groups that meet the eligibility criteria will be used in the data synthesis. To avoid problems with dependence between effect sizes we will apply robust standard errors (Hedges et al., 2010) and use the small sample adjustment to the estimator itself (Tipton, 2015). We will use the results in Tanner‐Smith and Tipton (2014) and Tipton (2015) to evaluate if there are enough studies for this method to consistently estimate the standard errors. See Section 3.3.11 below for more details about the data synthesis. If there are not enough studies, we will use a synthetic effect size (the average) to avoid dependence between effect sizes. This method provides an unbiased estimate of the mean effect size parameter but overestimates the standard error. Random effects models applied when synthetic effect sizes are involved actually perform better in terms of standard errors than do fixed effects models (Hedges, 2007a). However, tests of heterogeneity when synthetic effect sizes are included are rejected less often than nominal.

If pooling is not appropriate (e.g., the multiple intervention groups include the same individuals), only one intervention group will be coded and compared to the control group to avoid overlapping samples. The choice of which estimate to include will be based on our risk of bias assessment. We will choose the estimate that we judge to have the least risk of bias (primarily, confounding bias and in case of equal scoring the missing outcome data domain will be used).

##### Multiple studies using the same sample of data

In some cases, several studies may have used the same sample of data or some studies may have used only a subset of a sample used in another study. We will review all such studies, but in the meta‐analysis we will only include one estimate of the effect from each sample of data. This will be done to avoid dependencies between the ‘observations’ (i.e., the estimates of the effect) in the meta‐analysis. The choice of which estimate to include will be based on our risk of bias assessment of the studies. We will choose the estimate from the study that we judge to have the least risk of bias (primarily, confounding bias). If two (or more) studies are judged to have the same risk of bias and one of the studies (or more) uses a subset of a sample used in another study (or studies) we will include the study using the full set of participants. Likewise, in the case where a study is continuously recruiting participants and publishes data first with some of the participants and then a second publication includes all of the previous participants plus some newly recruited ones, we will use the latest study if the risk of bias judgement is the same.

##### Multiple time points

When the results are measured at multiple time points, each outcome at each time point will be analysed in a separate meta‐analysis with other comparable studies taking measurements at a similar time point. As a general guideline, these will be grouped together as stated in Section 3.1.4. However, should the studies provide viable reasons for an adjusted choice of relevant and meaningful duration intervals for the analysis of outcomes, we will adjust the grouping.

##### Multiple conceptually similar outcomes

Meta‐analysis of outcomes will be conducted on each metric (as outlined in Section 3.1.4) separately. If there are multiple estimates of effects regarding the same/similar outcome (e.g., resilience measured with both the CDRISC and the RSA measures), we will extract (and report) all outcomes, but in the meta‐analysis we will include one measure. We will include the most validated, best recognised, or most frequently used measures in the analysis.

#### 3.3.8 Dealing with missing data

Missing data and attrition rates will be assessed in the included studies; see Section 3.3.4. Where studies have missing summary data, such as missing standard deviations, the review authors will request this information from the principal investigators. If no information is yielded, we will derive these where possible from *F*‐ratios, *t*‐values, chi‐squared values and correlation coefficients using the methods suggested by Lipsey and Wilson (2001). If missing summary data cannot be derived, the study results will be reported in as much detail as possible.

#### 3.3.9 Assessment of heterogeneity

Heterogeneity amongst primary outcome studies will be assessed with chi‐squared (*Q*) test, and the *I* ^2^, and *τ* ^2^ statistics (Higgins, 2003). Any interpretation of the chi‐squared test will be made cautiously on account of its low statistical power.

#### 3.3.10 Assessment of reporting biases

Reporting bias refers to both publication bias and selective reporting of outcome data and results. Here, we state how we will assess publication bias.

We will use funnel plots for information about possible publication bias if we find a sufficient number of studies, that is, at least 10 (Higgins & Green, 2011). However, asymmetric funnel plots are not necessarily caused by publication bias (and publication bias does not necessarily cause asymmetry in a funnel plot). In general, asymmetry is a sign of small study effects, of which there can be many causes beside publication bias (Sterne et al., 2005).

Instead of trying to interpret the funnel plots as direct evidence of publication bias, or the lack thereof, we will perform sensitivity analyses for publication bias in meta‐analyses as suggested by Mathur and VanderWeele (2020). The method suggested by Mathur and VanderWeele (2020) gives a value of how large ratios of publication probabilities (i.e., the likelihood of affirmative results to be published relative to non‐affirmative results) would have to be to alter the results and therefore indicate how robust the meta‐analysis is to publication bias.

#### 3.3.11 Data synthesis

The proposed project will follow standard procedures for conducting systematic reviews using meta‐analysis techniques.

All follow‐up durations reported in the primary studies will be recorded, and we will do separate analyses for short‐term, medium term and long‐term outcomes.

The overall data synthesis will be conducted where effect sizes are available or can be calculated, and where studies are similar in terms of the outcome measured. Meta‐analysis of outcomes will be conducted on each metric (as outlined in Section 3.1.4) separately. As different computational methods may produce effect sizes that are not comparable, we will be transparent about all methods used in the primary studies (research design and statistical analysis strategies) and use caution when synthesising effect sizes. Special caution will be taken concerning studies using RDD to estimate the treatment effect. In sharp RDDs, a threshold of a (non‐manipulable) forcing/running variable determines which employees receive a treatment and which do not, that is, the design is similar to an RCT in the sense that the random sequence determining treatment assignment can be seen as a running variable (Lee & Lemieux, 2008). In contrast ‘fuzzy’ RDDs is a special type of IV. In ‘fuzzy’ RDDs, being on one side of a threshold only makes it more likely that a student ends up in the treatment or control group, and the threshold is used as an instrument to estimate local average treatment effects (Angrist & Pischke, 2009; Imbens & Lemieux, 2008). That is, fuzzy RDD is a form of IV analysis, which we will exclude due to the comparability issues mentioned earlier. Sharp RDDs will be included, but, as the effects may be estimated on a very ‘local’ sample close to a threshold, they may be subject to a separate analysis depending on the comparability to samples from other studies. We will in any case check the sensitivity of our results to the inclusion of RDD studies. In addition, we will discuss the limitation in generalisation of results obtained from these types of studies.

When the effect sizes used in the data synthesis are odds ratios, they will be log transformed before being analysed. The reason is that ratio summary statistics all have the common feature that the lowest value that they can take is 0, that the value 1 corresponds with no intervention effect, and the highest value that an odds ratio can ever take is infinity. This number scale is not symmetric. The log transformation makes the scale symmetric: the log of 0 is minus infinity, the log of 1 is zero, and the log of infinity is infinity.

Studies that have been coded with a Critical risk of bias will not be included in the data synthesis.

As the intervention deals with diverse populations of participants (from different occupations, facing different exposures, etc.), and we therefore expect heterogeneity amongst primary study outcomes, all analyses of the overall effect will be inverse variance weighted using random effects statistical models that incorporate both the sampling variance and between study variance components into the study level weights. Random effects weighted mean effect sizes will be calculated using 95% confidence intervals, and we will provide a graphical display (forest plot) of effect sizes. Graphical displays for meta‐analysis performed on ratio scales sometimes use a log scale, as the confidence intervals then appear symmetric. This is however not the case for the software Revman 5.4.1 which we plan to use in this review (If we apply robust variance estimation [RVE], the analysis will be conducted in Stata or R as RVE is not implemented in Revman 5.4.1). The graphical displays using odds ratios and the mean effect size will be reported as an odds ratio. Heterogeneity amongst primary outcome studies will be assessed with chi‐squared (*Q*) test, and the *I* ^2^, and *τ* ^2^ statistics (Higgins, 2003). Any interpretation of the chi‐squared test will be made cautiously on account of its low statistical power.

In addition to 95% confidence intervals, we will report 95% prediction intervals.

As meta‐analyses of 10 or less studies have been shown to produce confidence intervals that does not have the correct coverage, we will in addition report a confidence interval based on an alternative variance estimator (Zejnullahi & Hedges, 2023). The simulations reported in Zejnullahi & Hedges (2023) consider coverage probability, and so forth, using one of three conventional methods (DL, Hartung, HKSJ) and one of three robust variance methods (two with degrees of freedom adjustments as well) under various scenarios. Depending on the scenario (magnitude of effect size, number of studies, true heterogeneity and difference in sample sizes) one can choose a method that either underperform or overperform. However, the authors clearly recommend the so‐called HC3 estimator (and the adjusted HC3 if the number of studies is two) and we will report this variance estimator in addition to the conventional (DL) estimator when the number of studies are 2 <*k* ≤10. Note also that the authors report that they find that plugging in the DL estimate of the heterogeneity variance parameter, *τ* ^2^, performed the best.

For subsequent analyses of moderator variables that may contribute to systematic variations, we will use the mixed‐effects regression model if there is a sufficient number of studies. This model is appropriate if a predictor explaining some between‐studies variation is available, but there is a need to account for the remaining uncertainty (Hedges & Pigott, 2004; Konstantopoulos, 2006).

Studies may provide results separated by, for example, age and/or gender. We will include results for all age and gender groups. To take into account the dependence between such multiple effect sizes from the same study, we will apply the RVE approach (Hedges et al., 2010). An important feature of this analysis is that the results are valid regardless of the weights used. For efficiency purposes, we will calculate the weights using a method proposed by Hedges et al. (2010). This method assumes a simple random‐effects model in which study average effect sizes vary across studies (*τ* ^2^) and the effect sizes within each study are equi correlated (*ρ*). The method is approximately efficient, since it uses approximate inverse‐variance weights: they are approximate given that *ρ* is, in fact, unknown and the correlation structure may be more complex. We will calculate weights using estimates of *τ* ^2^, setting *ρ* = 0.80 and conduct sensitivity tests using a variety of *ρ* values; to assess if the general results and estimates of the heterogeneity is robust to the choice of *ρ*. We will use the small sample adjustment to the residuals used in RVE as proposed by Bell and McCaffrey (2002) and extended by McCaffrey et al. (2001) and Tipton (2015). We will use the Satterthwaite degrees of freedom (Satterthwaite, 1946) for tests as proposed by Bell and McCaffrey (2002) and extended by Tipton (2015). We will use the guidelines provided in Tanner‐Smith and Tipton (2014) to evaluate if there are enough studies for this method to consistently estimate the standard errors.

If there is not a sufficient number of studies to use RVE we will conduct a data synthesis where we use a synthetic effect size (the average) to avoid dependence between effect sizes.

#### 3.3.12 Subgroup analysis and investigation of heterogeneity

We will investigate the following factors with the aim of explaining potential observed heterogeneity: Study‐level summaries of the participant characteristics gender (e.g., studies considering a specific gender or studies where separate effects for men/women are available), age (e.g., 40‐year‐old and younger/41‐year‐old and older), and occupation (e.g., studies considering a specific occupation or studies where separate effects for different occupations are available). In addition, we will investigate other programme characteristics such as theoretical foundation of the programme (e.g., CBT‐based or mindfulness‐based), setting of resilience interventions (e.g., group setting or individual setting) and intensity (e.g., low or high intensity measured by hours of training). Note that the cut off age of 40 years is a bit arbitrary (halfway through life) and if the included studies offers a possibility of analysing another cut off age, we will use this.

If the number of included studies is sufficient and given there is variation in the covariates (age, gender, occupation and programme characteristics), we will perform moderator analyses (multiple meta‐regression using the mixed model) to explore how observed variables are related to heterogeneity.

If there is a sufficient number of studies, we will apply the RVE approach and use approximate inverse variance weights calculated using a method proposed by Hedges et al. (2010). This technique calculates standard errors using an empirical estimate of the variance: it does not require any assumptions regarding the distribution of the effect size estimates. The assumptions that are required to meet the regularity conditions are minimal and generally met in practice. This more robust technique is beneficial because it takes into account the possible correlation between effect sizes separated by the covariates within the same study (e.g., occupation or gender separated effects) and allows all the effect size estimates to be included in meta‐regression. We will calculate weights using estimates of *τ* ^2^, setting *ρ* = 0.80 and conduct sensitivity tests using a variety of *ρ* values; to assess if the general results are robust to the choice of *ρ*. We will use the small sample adjustment to the residuals used in RVE and the Satterthwaite degrees of freedom (Satterthwaite, 1946) for tests (Tipton, 2015). The results in Tipton (2015) suggest that the degrees of freedom depend on not only the number of studies but also on the type of covariates included in the meta‐regression. The degrees of freedom can be small, even when the number of studies is large, if a covariate is highly unbalanced or a covariate with very high leverage is included. The degrees of freedom will vary from coefficient to coefficient. The corrections to the degrees of freedom enable us to assess when the RVE method performs well. As suggested by Tanner‐Smith and Tipton (2014) and Tipton (2015) if the degrees of freedom are smaller than four, the RVE results should not be trusted. We will report 95% confidence intervals for regression parameters. We will estimate the correlations between the covariates and consider the possibility of confounding. Conclusions from meta‐regression analysis will be cautiously drawn and will not solely be based on significance tests. The magnitude of the coefficients and width of the confidence intervals will be taken into account as well. Otherwise, single factor subgroup analysis will be performed if there are at least two studies in each subgroup. The assessment of any difference between subgroups will be based on 95% confidence intervals. Interpretation of relationships will be cautious, as they are based on subdivision of studies and indirect comparisons. In general, the strength of inference regarding differences in treatment effects amongst subgroups is controversial. However, making inferences about different effect sizes amongst subgroups on the basis of between‐study differences entails a higher risk compared to inferences made on the basis of within study differences (see Schandelmaier et al., 2020). We will therefore use within study differences where possible.

We will also consider the degree of consistence of differences, as making inferences about different effect sizes amongst subgroups entails a higher risk when the difference is not consistent within the studies (Schandelmaier et al., 2020).

#### 3.3.13 Sensitivity analysis

Sensitivity analysis will be carried out by restricting the meta‐analysis to a subset of all studies included in the original meta‐analysis and will be used to evaluate whether the pooled effect sizes are robust across components of risk of bias. We will consider sensitivity analysis for each domain of the risk of bias checklists and restrict the analysis to studies with a low risk of bias.

Sensitivity analyses with regard to research design and statistical analysis strategies in the primary studies will be an important element of the analysis to ensure that different methods produce consistent results.

#### 3.3.14 Treatment of qualitative research

We do not plan to include qualitative research.

#### 3.3.15 Summary of findings and assessment of the certainty of the evidence

The GRADE (Grades of Recommendation, Assessment, Development, and Evaluation) system will be used to assess the certainty of the body of evidence as it relates to the studies that contribute data to the meta‐analyses for the prespecified outcomes (Guyatt et al., 2013). The system classifies certainty of evidence as high, moderate, low, or very low (Guyatt et al., 2013). Five of the eight criteria proposed in the GRADE method have the potential to decrease one's confidence in the correctness of the effect estimates: risk of bias, inconsistency of results across studies, indirectness of evidence, imprecision, and publication bias. Three further criteria are proposed that have the potential to increase this confidence: a large magnitude of effect with no plausible confounders, a dose–response gradient, and a conclusion that all plausible residual confounding would further support inferences regarding treatment effect. GRADE proposes these three criteria should be considered particularly in observational studies. (Guyatt et al., 2013). We will justify all decisions to downgrade or upgrade the certainty of outcomes, and make comments to aid readers' understanding of the review where necessary.

The outcomes will be graded as follows.

- High certainty: further research is very unlikely to change our confidence in the effect estimate.
- Moderate certainty: further research is likely to have an important impact on our confidence in the effect estimate and may change the estimate.
- Low certainty: further research is very likely to have an important impact on our confidence in the effect estimate and may change the estimate.
- Very low certainty: we are very uncertain about the effect estimate.

We will produce a summary of findings table, presenting the overall quality of the body of evidence according to GRADE criteria for the mental health outcomes.

## CONTRIBUTIONS OF AUTHORS

- Content: Trine Filges and Geir Smedslund.
- Systematic review methods: Trine Filges, Anja Bondebjerg, Malene Wallach Kildemoes, and Geir Smedslund.
- Statistical analysis: Trine Filges and Geir Smedslund.
- Information retrieval: Elizabeth Bengtsen.

## DECLARATIONS OF INTEREST

None.

## PRELIMINARY TIMEFRAME

We plan to submit the review within 18 months of protocol publication.

## PLANS FOR UPDATING THIS REVIEW

Trine Filges will be responsible for updates.

## DATA AND ANALYTIC CODE

Data coding sheets and analytic codes will be submitted as supplementary material.

## SOURCES OF SUPPORT

### Internal sources

VIVE Campbell, Denmark.

### External sources

None.

## Supporting information

Supporting information.
